# Comment on “Predicting mental health–related sick leave using stress check data: development and validation of a workplace risk model”

**DOI:** 10.1093/joccuh/uiag020

**Published:** 2026-04-28

**Authors:** Kei Muroi, Shotaro Doki

**Affiliations:** International Institute for Integrative Sleep Medicine (WPI-IIIS), Tsukuba Institute for Advanced Research (TIAR), University of Tsukuba, 1-1-1 Tennodai, Tsukuba, Ibaraki 305-8575, Japan; Institute of Medicine, University of Tsukuba, Tsukuba, Japan; CEO, Minds in Co., Ltd., Chuo-ku, Japan

**Keywords:** Brief Job Stress Questionnaire, sickness absence, prediction model, area under the curve, precision–recall, machine learning

Dear Editor

We read with great interest the article by Inoue et al reporting a mixed-effects logistic regression model predicting mental health–related sickness absence (≥1 month) using Brief Job Stress Questionnaire (BJSQ) data.^1^ A recent study by Iwasaki et al,^2^ published in *Scientific Reports*, evaluated machine-learning models for predicting long-term sickness absence due to mental disorders (≥90 days; ICD-10 F00–F99) using BJSQ variables among Japanese local government employees. They reported a best-case average precision of 0.040. The contrasting conclusions of these 2 studies warrant methodological clarification based on the following 3 points.

First, the outcome definitions differ substantially. Inoue et al used a ≥1-month threshold aligned with Japan’s national Occupational Safety and Health Survey, whereas Iwasaki et al required ≥90 days confirmed by a physician’s certificate. These thresholds capture different severities of absence, and are not directly comparable. The key methodological differences between the 2 studies are summarized in Table 1.

**Table 1:** Key methodological differences between 2 studies predicting mental health–related sickness absence using BJSQ data.

Feature	Inoue et al^1^	Iwasaki et al^2^
**Population**	Employees in 14 affiliated Japanese companies	Japanese local government employees
**Sample size**	~109 000 person-years	~231 000 person-years
**Outcome definition**	Mental health–related sickness absence ≥1 month	Sickness absence ≥90 days due to ICD-10 F00–F99
**Data structure**	Annual repeated observations per worker (person-year dataset)	Annual questionnaire data linked with sickness absence records
**Prediction model**	Mixed-effects logistic regression	Machine-learning models (random forest, gradient boosting, SVM, neural network)
**Predictors**	Selected BJSQ subscales + age, sex, occupation	All 57 BJSQ items + sociodemographic variables
**Outcome prevalence**	0.8% (development); 0.26% (validation)	0.53%
**Primary evaluation metric**	AUC (ROC curve)	Average precision (precision–recall curve)
**Reported performance**	AUC = 0.819 (validation)	Average precision = 0.040 (best model)
**Within-person correlation handling**	Random intercepts for company and year; individual-level random intercept in sensitivity analysis only	Stratified 70/30 split; no worker-level partitioning

Abbreviations: AUC, area under the ROC curve; BJSQ, Brief Job Stress Questionnaire; ICD-10, International Classification of Diseases, 10th Revision; ROC, receiver operating characteristic; SVM, support vector machine.

Second, the choice of evaluation metric explains much of the apparent discrepancy. Area under the receiver operating characteristic curve (AUC) measures rank discrimination across all thresholds, but is insensitive to outcome prevalence.^3^ When the prevalence is low, a high AUC is achievable even when the positive predictive value remains poor. Inoue et al reported precision–recall metrics, as shown in Table 4 of their publication; at the F1-optimized cutoff of 0.023, the precision (positive predictive value) was 2.9% in development and 1.3% in validation, with recall (sensitivity) of 26.5% and 19.3%, respectively.^1^ However, the Abstract and Key points emphasize AUC = 0.819, while the implication of precision below 2% (meaning fewer than 1 in 50 workers flagged as high-risk actually experienced sickness absence) receives limited discussion.^4^ The operational significance of the threshold choice is further illustrated by the Youden-optimized cutoff of 0.008, at which approximately 29% of the workforce would be flagged for follow-up (calculated by the authors based on the reported data). Occupational health practitioners selecting prediction tools for workplace implementation need metrics that reflect the operational screening yield, not only rank discrimination. Rather than requiring individual follow-up for all flagged workers, the model’s value may lie in supporting prioritization and guiding lower-burden approaches such as self-monitoring tools or group-level interventions, which are feasible within typical occupational health staffing in Japanese workplaces.

Third, both studies used repeated annual observations per worker without explicitly adjusting for within-person correlations. Inoue et al included random intercepts for company and year but added an individual-level random intercept only in a sensitivity analysis. Iwasaki et al applied a stratified 70/30 train/test split without worker-level partitioning. Assigning all observations from a given individual to either training or validation data would provide more conservative estimates of the generalization performance in future studies.

These considerations do not diminish the practical contribution of Inoue et al, who provided a reproducible prediction workflow within Japan’s existing Stress Check framework. We encourage the authors to consider reporting precision–recall metrics prominently in future communications on this model. As Japan’s Stress Check program is under discussion for expansion to smaller workplaces,^5^ standardization of reporting practices, including explicit specification of outcome thresholds, presentation of precision–recall metrics alongside AUC in abstracts, and worker-level data partitioning, would support a realistic appraisal of model utility and inform evidence-based policies on occupational mental health screening.
